# The Threatful Self: Midbrain Functional Connectivity to Cortical Midline and Parietal Regions During Subliminal Trauma-Related Processing in PTSD

**DOI:** 10.1177/2470547019871369

**Published:** 2019-09-05

**Authors:** Braeden A. Terpou, Maria Densmore, Jean Théberge, Janine Thome, Paul Frewen, Margaret C. McKinnon, Ruth A. Lanius

**Affiliations:** 1 Department of Neuroscience, Western University, London, ON, Canada; 2 Department of Psychiatry, Western University, London, ON, Canada; 3 Imaging Division, Lawson Health Research Institute, London, ON, Canada; 4 Department of Medical Biophysics, Western University, London, ON, Canada; 5 Department of Theoretical Neuroscience, Central Institute of Mental Health Mannheim, Medical Faculty Mannheim, Heidelberg University, Heidelberg, BW, Germany; 6 Department of Psychology, Western University, London, ON, Canada; 7 Mood Disorders Program, St. Joseph’s Healthcare, Hamilton, ON, Canada; 8 Department of Psychiatry and Behavioural Neurosciences, McMaster University, Hamilton, ON, Canada; 9 Homewood Research Institute, Guelph, ON, Canada

**Keywords:** post-traumatic stress disorder, subliminal, periaqueductal gray, default-mode network, midbrain, psycho-physiological interaction

## Abstract

**Background:**

The innate alarm system consists of a subcortical network of interconnected midbrain, lower brainstem, and thalamic nuclei, which together mediate the detection of evolutionarily-relevant stimuli. The periaqueductal gray is a midbrain structure innervated by the innate alarm system that coordinates the expression of defensive states following threat detection. In participants with post-traumatic stress disorder, the periaqueductal gray displays overactivation during the subliminal presentation of trauma-related stimuli as well as altered resting-state functional connectivity. Aberrant functional connectivity is also reported in post-traumatic stress disorder for the default-mode network, a large-scale brain network recruited during self-referential processing and autobiographical memory. Here, research lacks investigation on the extent to which functional interactions are displayed between the midbrain and the large-scale cortical networks in post-traumatic stress disorder.

**Methods:**

Using a subliminal threat presentation paradigm, we investigated psycho-physiological interactions during functional neuroimaging in participants with post-traumatic stress disorder (n = 26) and healthy control subjects (n = 20). Functional connectivity of the periaqueductal gray was investigated across the whole-brain of each participant during subliminal exposure to trauma-related and neutral word stimuli.

**Results:**

As compared to controls during subliminal threat presentation, the post-traumatic stress disorder group showed significantly greater periaqueductal gray functional connectivity with regions of the default-mode network (i.e., angular gyrus, precuneus, superior frontal gyrus). Moreover, multiple regression analyses revealed that the functional connectivity between the periaqueductal gray and the regions of the default-mode network correlated positively to symptoms of avoidance and state dissociation in post-traumatic stress disorder.

**Conclusion:**

Given that the periaqueductal gray engages the expression of defensive states, stronger midbrain functional coupling with the default-mode network may have clinical implications to self-referential and trauma-related processing in participants with post-traumatic stress disorder.

## Introduction

The innate alarm system (IAS) refers to a subcortical network of interconnected midbrain, lower brainstem, and thalamic nuclei, which together mediate the detection of evolutionarily-relevant stimuli in the environment.^1^ The IAS is centralized on the superior colliculus, a midbrain structure that processes and transmits multisensory information. For visual stimuli, projections from the retina are relayed through the superior colliculus and the pulvinar of the thalamus and directed toward frontolimbic neural circuits.^2^ Given its rapid transmission and bypass of primary sensory cortices, visual information processed by the IAS is represented crudely.^1^ This hastened transmission of threat stimuli, however, confers an evolutionary advantage to the individual, with the IAS postulated to function during subliminal exposure.^3^ Subliminal exposure refers to sensory information that is not perceived consciously but can nonetheless generate an increase in activation of threat detection circuits and, as a corollary, neural systems involved in defensive responding.^1,4^

The periaqueductal gray (PAG) is a midbrain structure innervated by the superior colliculus, in addition to other brainstem nuclei, the spinal cord, the amygdala, the hypothalamus, and the cortex and is thus well positioned to coordinate defensive responses to a perceived threat.^5–7^ Defensive responses refer to a set of behavioral states that are engaged through the excitation or the inhibition of the sympathetic nervous system, as well as through the expression of opioid- or endocannabinoid-mediated analgesia.^8,9^ Behaviorally, defensive responses may take the form of an active (i.e., fight and flight) or of a passive state (i.e., tonic immobility and shutdown) and their expression is dependent on the context and the level of threat perceived.^10^ In rodents, electrical stimulation of the PAG induces elevated levels of fighting and/or fleeing that are coincident with increases in heart rate, core body temperature, and blood pressure.^11,12^ These rodent findings corroborate human studies employing functional magnetic resonance imaging (fMRI) during threat anticipation paradigms to model brain activation as a function of the imminence of a threat encounter.^13,14^ In these studies, Mobbs et al. have shown that as the distance between an individual and a perceived threat decreases, there is a concordant shift in brain activation from a pattern of top-down, or ventromedial prefrontal-mediated, to a pattern of bottom-up processing.^13^ Specifically, as the imminence of danger increases, a pattern of bottom-up processing involving increased activation of the locus coeruleus, the PAG, and the amygdala is observed. These increases in activation have been interpreted as evidence for the predominance of evolutionarily-conserved, subcortical systems of response during experiences of imminent threat, that contrast sharply with the more cognitive, top-down systems of response observed when threat is perceived at a distance.^14^ Critically, the degree to which the PAG is activated in response to threat stimuli may increase as a function of prior lifetime experiences and, in particular, of trauma exposure.^15^

Post-traumatic stress disorder (PTSD) is a mental disorder characterized by hypervigilance, hyperarousal, and, at times, dissociative symptoms following exposure to a traumatic experience.^16^ Often, exposure to a traumatic event can promote an attentional threat bias, or threat sensitization, whereby negatively valenced stimuli are processed preferentially, leading to exaggerated PTSD symptoms.^17–19^ This attentional bias is thought to be the product of the overactivation of threat detection circuitry and, in particular, the IAS.^20^ Notably, several structures associated with the IAS display overactivation during the presentation of fear- or trauma-related material in PTSD, including the amygdala,^21–23^ the parahippocampal gyrus,^24,25^ the lower brainstem,^26,27^ and the PAG.^26,28,29^ Critically, this pattern of neural response emerges under conditions of subliminal and of supraliminal presentation.^21,26,27^ In particular, a recent study by Terpou et al.^29^ revealed a cluster of significantly greater activation of the PAG, as compared to controls, in participants with PTSD during the subliminal presentation of trauma-related word stimuli—to which the present report builds on these findings.

In addition to increased activation during threat detection, the PAG demonstrates aberrant functional characteristics in individuals with PTSD during rest, where PTSD symptoms are present not only during threat- or trauma-related processing but also during baseline conditions.^30–32^ Here, the PAG exhibits increased resting-state functional connectivity with cortical regions associated with environmental monitoring and with autonomic nervous system regulation in individuals with PTSD as compared to healthy controls.^33^ These findings suggest a strong association between subcortical systems involved in defensive responding and high-order, cognitive circuits of the brain in PTSD.^20^ To ascertain the directionality of these subcortical–cortical interactions, Nicholson et al.^34^ employed dynamic causal modeling of resting-state fMRI in a group of participants with and without PTSD. The results of this study revealed that, as compared to controls, the PTSD group had a stronger pattern of directed connectivity extending from the PAG toward the amygdala and the ventromedial prefrontal cortices. Taken together, these findings provide evidence for a bottom-up or PAG-mediated pattern of neuronal connectivity in PTSD.

The increased functional connectivity directed from the PAG toward the cortex in PTSD may interfere significantly with the function of large-scale intrinsic connectivity networks. An intrinsic connectivity network (ICN) is a neurocognitive network of brain regions which displays high functional connectivity between network nodes.^35^ The default-mode network (DMN) is a task-negative ICN active during self-referential processing, internal cognition, and autobiographical memory retrieval.^36^ The DMN contains a series of functional hubs that extend along the mid-line of the brain and include the medial prefrontal, posterior cingulate, and posterior parietal cortices.^35,37^ Critically, individuals with PTSD show reduced resting-state functional connectivity between anterior prefrontal (i.e., ventromedial prefrontal, anterior cingulate) and posterior parietal nodes (i.e., precuneus, posterior cingulate) as compared with controls, and these reductions correlate to symptom severity.^38–41^ Here, aberrant DMN connectivity is thought to contribute to clinical disturbances in self-related processing among individuals with PTSD, which may include altered self-perceptions of body state and of emotional and perceptual experiences.^42–44^ Disturbances in self-related processing are associated more strongly with the dissociative subtype of PTSD, which is identified by greater illness severity and the presence of supplementary dissociative symptoms (i.e., depersonalization, derealization) during threat- or trauma-related stimulus exposure.^45–47^

The research summarized above highlights the importance of threat detection systems and features the influential role the PAG serves in responding to threat. In addition, we discussed the function of the DMN and the atypical characteristics that are displayed within this network in PTSD. Despite a preponderance of evidence suggesting a strong influence of bottom-up processes, research rarely investigates functional connectivity patterns between the midbrain and large-scale cortical networks. Accordingly, our aim was to investigate the functional connectivity displayed by the PAG in participants with PTSD and control subjects during subliminal threat processing. The present report extends on a previous study that revealed greater activation of the PAG in PTSD as compared with controls during subliminal trauma-related word exposure.^29^ Psycho-physiological interactions are conducted here to analyze group-level differences in the functional connectivity exhibited by the PAG seed that is reported in the previous study during subliminal presentation. We predicted that the PTSD group will show increased PAG functional connectivity with the DMN during subliminal threat exposure as a result of co-activation of self-referential and threat processing systems. The DMN is activated during self-referential processing; we hypothesize that the onset of trauma-related cues to participants with PTSD will stimulate these self-referential systems as well as the PAG to mediate the fear-inducing effects. The coengagement of these systems is thought to produce a strong functional relatedness to be determined in this study.

## Methods

### Participants

The study was approved by the Health Sciences Research Ethics Board of Western University and adhered to the standards set forth by the Tri-Council Policy. The study included forty-six English-speaking participants recruited by the London Health Services Centre via referrals from physicians, community clinics, mental health professionals, and advertisements. In total, twenty-six participants met the criteria for a primary diagnosis of PTSD, and the remaining twenty participants were included as healthy, non-trauma-exposed controls. Written and informed consent was provided by all participants. The analyses discussed in this article are novel; however, the data generated on this sample are analyzed in our other published works.^27,29,48,49^

The exclusion criteria for participation in the study included incompatibilities with the scanning requirements, previous neurologic and development illness, comorbid schizophrenia or bipolar disorder, alcohol or substance abuse within six months prior to scanning, a history of head trauma, or pregnancy during the time of the scan. Diagnoses were determined using the Clinician Administered PTSD Scale (CAPS)^50^ and confirmed by a Structured Clinical Interview for DSM-IV Axis-I disorders.^51^ Control subjects were permitted if they did not meet any current or lifetime criteria for a psychiatric disorder, and participants with PTSD were medication free for at least six weeks prior to scanning. In addition to the diagnostic inventories, participants completed a battery of questionnaires prior to scanning, which included the Beck’s Depression Inventory (BDI),^52^ the Childhood Trauma Questionnaire (CTQ),^53^ and the Multiscale Dissociation Inventory (MDI).^54^ Whereas twenty-three of the twenty-six participants diagnosed with PTSD had experienced childhood interpersonal trauma as their trauma origin, the remaining three of the twenty-six participants had experienced a personal threat of life or had witnessed a violent death. None of the participants in the current sample were diagnosed with PTSD related to military trauma. After fMRI scanning was completed, participants were administered state-related inventories, including the State-Trait Anxiety Inventory (STAI),^55^ the Responses to Script-Driven Imagery (RSDI),^56^ and the Clinician Administered Dissociative States Scale (CADSS).^57^

### Experimental Task

**Figure 1.**
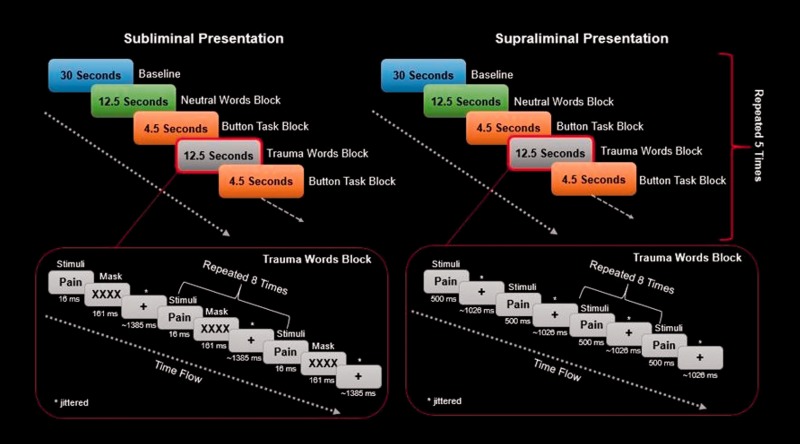
An illustration of the subliminal-supraliminal threat presentation paradigm. Stimuli had one subliminal and one supraliminal presentation session over two consecutive sessions that were counterbalanced across subjects and involved a 2-min rest period between the sessions. Stimuli represented both threat as well as neutral cues, presented in a pseudo-randomized block design (i.e., pseudo-randomized since NWs were not to follow trauma-related or fearful stimuli). Each presentation block was repeated five times in a fixed order to the participant. Blocks consisted of eight repetitions of stimuli with either a subliminal or a supraliminal display. Subliminal stimuli were presented for 16 ms and separated by a jittered interstimulus interval that varied in duration from 823 to 1823 ms and were followed by a mask. Supraliminal stimuli were presented for 500 ms and separated by a jittered interstimulus interval of 500 to 1500 ms. A button press task was implemented between presentation blocks to ensure sustained attention throughout the fMRI scanning session. Finally, each run was preceded by a 30-s rest period that was used as an implicit baseline for subsequent statistical analyses.

The paradigm and psychophysical thresholds used were based on previously published methods.^26,27,58^ Stimuli had a subliminal and a supraliminal display session over two consecutive sessions that were counterbalanced across subjects and involved a two-minute rest period between. Stimuli represented both threat (fearful faces (FF) and individualized trauma-related words (TW)) and neutral (neutral faces (NF) and neutral words (NW)) cues, presented in a pseudo-randomized block design. Word-related stimuli were subject specific, with TWs generated in reference to a traumatic memory or, in the case of controls, an aversive experience. Neutral words were selected had they not elicited a strong positive or negative reaction during pre-scan exposure to the word. Trauma-related and NWs were matched for syllable and for letter length. For a more detailed description of the subliminal-supraliminal threat protocol, please refer to Figure 1.

### fMRI Data Acquisition

Functional images were collected using a 3.0 T whole-body MRI scanner (Siemens Biograph mMR, Siemens Medical Solutions, Erlangen, Germany) with a 32-channel phased-array head coil. T1-weighted anatomical images were collected with 1 mm isotropic resolution (MP-RAGE, TR/TE/TI = 2300 ms/2.98 ms/900 ms, FA 9°, FOV = 256 mm × 240 mm × 192 mm, acceleration factor = 4, total acquisition time = 192 s). For blood-oxygen-level dependent (BOLD) fMRI, transverse imaging slices covering the whole-brain were prescribed parallel to the anterior commissure-posterior commissure line. Functional data were acquired using a gradient echo planar imaging sequence (single-shot, blipped) with an interleaved slice acquisition order and tridimensional prospective acquisition correction (3D PACE) and an isotropic resolution of 2 mm [(FOV = 192 mm × 192 mm × 128 mm (94 × 94 matrix, 64 slices)), TR/TE = 3000 ms/20 ms, FA = 90° (FOV = Field of View, TR = Repetition Time, TE = Echo Time, FA = Flip Angle)].

Data were analyzed using Statistical Parametric Mapping (SPM12, Wellcome Trust Centre for Neuroimaging, London, UK: http://www.fil.ion.ucl.ac.uk/sp) within MATLAB 9.2 (R2017a, Mathworks Inc., MA). A breakdown of the preprocessing steps for whole-brain and the spatially unbiased infratentorial template (SUIT)^59,60^ toolbox are provided in the Supplemental Materials.

### Statistical Analyses

#### Within-Subject: Psychological Regressor

Within the first-level analyses, a fixed-effects general linear model was created for each subject with three main factors, each with two experimental levels (Factor 1: Group: PTSD, Control; Factor 2: Conscious Level: Subliminal, Supraliminal; Factor 3: Stimuli: Faces (FF, NF), Words (TW, NW)). The signal derived from the stimulus onsets were modeled as the convolution of the stimulus function to the default hemodynamic response function. The button press task, realignment parameters, and artifact detection regressor were included as regressors of no interest. The experimental conditions were used to generate contrasts between threat and neutral conditions for both subliminal and supraliminal presentation sessions (i.e., FF > NF, TW > NW). These contrasts were carried into the second-level for between-group analyses. The results from these subtraction analyses have been published by Terpou et al.^29^ and are restricted to the partial-brain space as offered by the SUIT toolbox. The SUIT toolbox improves the normalization procedure of the midbrain, lower brainstem, and cerebellum to offer greater resolution of these subcortical structures than can be afforded by whole-brain standards.^59,60^ In the previous study, significant results were generated only for the subliminal contrast of trauma-related minus neutral word exposure (Subliminal: TW > NW).^29^ As a result, the psycho-physiological interactions (PPIs) conducted here will focus on this experimental contrast as our psychological regressor of interest.

#### Within-Subject: Physiological Regressor

The physiological regressor for this study used the time course of the PAG that was informed by Terpou et al.^29^ The previous study was conducted on the same participant sample and paradigm and revealed greater PAG activation ([*x*: 0, *y*: −32, *z*: −11], *k* = 53, *p*-FWE = .013) in PTSD as compared to controls during the contrast of Subliminal: TW > NW. This study extracted the eigenvariate from the PAG by creating a spherical volume-of-interest of 6 mm centered on these coordinates to gather the seed time course of the PAG across all participants.

#### Between-Group: Psycho-physiological Interaction

The PPI interaction terms were obtained by deconvolving the BOLD signal of the PAG by the hemodynamic response function and then multiplying the deconvolved time series by the psychological variable (i.e., Subliminal: TW > NW). This generated a series of estimated interaction term parameters that were then reconvolved with the default hemodynamic response function. These interaction parameters were carried into the second-level for within- and between-group analyses. One- and two-sample t-tests were evaluated and reported at a significance threshold of *p*-FWE <.05*, k* > 10. A region-of-interest (ROI) analysis was also conducted using a DMN mask adopted from the accessible Functional Imaging in Neuropsychiatric Disorders Lab database that contained regions of the medial prefrontal, posterior cingulate, and posterior parietal cortices.^61^

#### Clinical Correlations

Multiple regression analyses were conducted within the PTSD group to determine whether clinical scores correlated with PAG functional connectivity. Interaction term parameters were correlated with symptom scores of reexperiencing (CAPS criterion B), avoidance (CAPS criterion C), negative alterations in cognition and mood (CAPS criterion D), dissociation (MDI), childhood trauma (CTQ), depressive symptomatology (BDI), as well as to state-related scores as measured by the STAI, RSDI, and CADSS.

## Results

As noted, these PPI analyses were guided by a previous study revealing group differences in activation of the PAG during Subliminal: TW > NW in participants with PTSD as compared to controls.^29^ However, the previous report failed to yield significant activation of the PAG for either group during supraliminal contrast conditions or the subliminal contrast of FF > NF. To this end, our analyses will focus on the subliminal display of trauma-related and NWs, specifically. All reported results for PPI analyses surpassed a significance threshold of *p*-FWE < .05*, k* > 10.

### Demographics and Clinical Measures

Independent t-tests conducted on demographic measures between the PTSD and the control group did not reveal significant differences. As expected for clinical measures, as compared to controls, participants with PTSD scored significantly higher on total scores for the CAPS, MDI, CTQ, and RSDI (Table 1).

**Table 1.** Clinical and demographic information.

Measure	PTSD (*N* = 26)	Healthy controls (*N* = 20)	χ^2^	t Test
	M ± SD	M ± SD	*p*	*p*
Years of age	38.8 ± 12.2	32.5 ± 11.6	.088	–
Sex (n)	Male = 11, Female = 15	Male = 10, Female = 10	.604	–
Employment status (n)	Employed = 18, Unemployed = 7	Employed = 17, Unemployed = 3	.297	–
CAPS Total	70.6 ± 11.9	.94 ± 2.9	–	<.001
CTQ—emotional abuse	14.5 ± 6.1	6.8 ± 3.1	–	<.001
CTQ—physical abuse	10.1 ± 6.4	5.7 ± 1.6	–	.004
CTQ—sexual abuse	13.4 ± 7.8	5.3 ± 1.1	–	<.001
CTQ—emotional neglect	13.5 ± 5.9	8.8 ± 4.2	–	.004
CTQ—physical neglect	10.2 ± 4.7	6.8 ± 2.7	–	.006
MDI total	58.8 ± 21.6	33.7 ± 3.8	–	<.001
MDI—depersonalization	7.8 ± 4.1	–	–	–
MDI—derealization	9.5 ± 4.5	–	–	–
MDI—dep./der.	8.7 ± 4.1	–	–	–
CADSS total	4.3 ± 2.6	–	–	–
STAI total	6.2 ± 2.5	–	–	–
RSDI total	4.1 ± 1.8	–	–	–
RSDI—distress	2.2 ± 0.9	1.0 ± 0.0	–	<.001
RSDI—reliving	2.0 ± 1.0	1.0 ± 0.0	–	.001
RSDI—avoidance thoughts	1.9 ± 0.8	1.1 ± 0.3	–	.001
Axis-I comorbidities (current [past]) frequency	Major depressive disorder (8 [9])			
	Dysthymic disorder (0 [3])			
	Agoraphobia w/o PD (3)			
	Social phobia (4)			
	Specific phobia (2)			
	OCD (1 [1])			
	Eating disorders (1 [1])			
	Somatoform disorder (6)			
	Lifetime alcohol abuse or dependence [16]			
	Lifetime substance abuse or dependence [7]			

Note: Age, sex, trait scores (CAPS Total, CTQ), MDI (Total, Dep, Der, Dep/Der), CADSS, STAI, RSDI (Total, Distress, Reliving, Avoidance Thoughts), and comorbidities for PTSD and control groups as mean values ± standard deviations. CAPS: Clinician Administered PTSD Scale; CTQ: Childhood Trauma Questionnaire; MDI: Multiscale Dissociation Inventory [Dep: Depersonalization Subscale; Der: Derealization Subscale; Dep/Der: Depersonalization and Derealization Subscales Averaged]; CADSS: Clinician Administered Dissociative States Scale; STAI: State-Trait Anxiety Inventory; RSDI: Responses to Script-Driven Imagery; PD: Panic Disorder; OCD: Obsessive-Compulsive Disorder.

### Within-Group PPI: PAG

The PPI analyses did not reveal significant results for the PAG within the control group for whole-brain or ROI analyses. The PTSD group, however, demonstrated significant whole-brain PAG functional connectivity with the medial segment of the superior frontal gyrus ([*x*: −2 *y*: 60 *z*: 12], *k* = 871, *p*-FWE = .003) as well as the right angular gyrus ([*x*: 54 *y*: −58 *z*: 34], *k* = 172, *p*-FWE = .021). Moreover, ROI analyses for the DMN mask yielded significant PAG connectivity with the medial segment of the superior frontal gyrus ([*x*: −2 *y*: 60 *z*: 12], *k* = 689, *p*-FWE = .001) as well as the precuneus ([*x*: 2 *y*: − 52 *z*: 32], *k* = 420, *p*-FWE = .017) in the PTSD group (Table 2).

**Table 2.** Within- and between-group differences in the psycho-physiological interaction of the PAG.

Contrast	LR	Region	*k*	*p*(FWE-cor)	*z*	MNI Coordinates		
						*x*	*y*	*z*
Subliminal TW > NW (WB)								
Control		None						
PTSD	L	Superior frontal gyrus	871	.003	5.46	−2	60	12
	R	Angular gyrus	172	.021	5.05	54	−58	34
Control > PTSD		None						
PTSD > Control		None						
Subliminal TW > NW (DMN ROI)								
Control		None						
PTSD	L	Superior frontal gyrus	689	.001	5.46	−2	60	12
		Medial segment of SFG	Of 689	.001	5.04	0	60	−2
	R	Precuneus	420	.017	4.38	2	−52	32
Control > PTSD		None						
PTSD > Control		Superior frontal gyrus	372	.003	4.75	0	60	−2
	L	Medial segment of SFG	Of 372	.007	4.55	−2	60	12
	R	Precuneus	192	.025	4.21	6	−52	30
		Anterior cingulate gyrus	69	.029	4.18	0	46	20

Note: Within- and between-group differences in BOLD functional connectivity between PTSD and controls within the subliminal threat presentation task. Reported results for whole-brain and ROI analyses are at a significance threshold of *p*-FWE < .05, *k* > 10. The contrast column lists the specific comparison of the experimental conditions. The hemisphere of the region (L/R), region, cluster size (*k*), significance (*p*(FWE)-cor), z-score (*z*), and MNI coordinates (*x, y, z*) of the peak coordinates are included as columns. PAG: periaqueductal gray; TW: trauma-related word stimulus; NW: neutral word stimulus; WB: whole-brain; DMN: default-mode network; ROI: region-of-interest; SFG: superior frontal gyrus.

### Between-Group PPI: PAG

Between-group findings did not yield significant results for greater PAG functional connectivity in the control group as compared to the PTSD group in whole-brain or ROI analyses. By contrast, results from the DMN ROI yielded significantly stronger PAG functional connectivity with the medial segment of the superior frontal gyrus ([*x*: 0 *y*: 60 *z*: −2], *k* = 372, *p*-FWE = .003), the right precuneus ([*x*: 6 *y*: −52 *z*: 30], *k* = 192, *p*-FWE = .025), and the anterior cingulate ([*x*: 0 *y*: 46 *z*: 20], *k* = 69, *p*-FWE = .029) in the PTSD group as compared to the control group (Table 2).

### Clinical Correlation PPI: PAG

Multiple regression analyses conducted between PTSD clinical scores and PAG functional connectivity yielded several significant results. A positive correlation was detected between state dissociation scores (CADSS) and functional connectivity exhibited between the PAG and the right middle frontal gyrus ([*x*: 34 *y*: 22 *z*: 46], *k* = 168, *p*-FWE = .037) in the PTSD group. Moreover, frequency/intensity scores of CAPS criterion B (re-experiencing) revealed a positive correlation with the functional connectivity between the PAG and the right posterior orbital gyrus ([*x*: 28 *y*: 28 *z*: −20], *k* = 45, *p*-FWE = .019). Finally, a positive association was revealed between CAPS criterion C (avoidance) symptom scores and PAG functional connectivity with the left middle temporal gyrus ([*x*: −60 *y*: −38 *z*: 2], *k* = 143, *p*-FWE = .044) in the PTSD group (Table 3). No significant results were generated for the multiple regression analysis for symptom measures of the CAPS criterion D subscale, MDI, CTQ, BDI, and the RSDI.

**Table 3.** Multiple regression of clinical scores with the psycho-physiological interaction of the PAG in PTSD.

Clinical Measure	Direction	LR	Region	*k*	*p*(FWE-cor)	*z*	MNI coordinates		
							*x*	*y*	*z*
Subliminal TW > NW (WB)									
CADSS	+	R	Middle frontal gyrus	168	.037	5.01	34	22	46
CAPS Criterion B Symptoms	+	R	Posterior orbital gyrus	45	.019	5.15	28	28	−20
CAPS Criterion C Subtotal	+	L	Middle temporal gyrus	143	.044	4.95	−60	−38	2

Note: Clinical correlations from the multiple regression analysis between clinical scores in the PTSD group and functional connectivity extending from the PAG during subliminal trauma-related word exposure greater than neutral word exposure. Reported results for whole-brain findings are at a significance threshold of *p*-FWE < .05, *k* > 10. The contrast column lists the specific inventory or questionnaire administered. The direction (+/−), hemisphere of the region (L/R), region, cluster size (*k*), significance (*p*(FWE)-cor), z-score (*z*), and MNI coordinates (*x, y, z*) of the peak are included as columns. CADSS: Clinician Administered Dissociative States Scale; CAPS: Clinician Administered PTSD Scale; PAG: periaqueductal gray; WB: whole-brain.

## Discussion

### Overview

**Figure 2.**
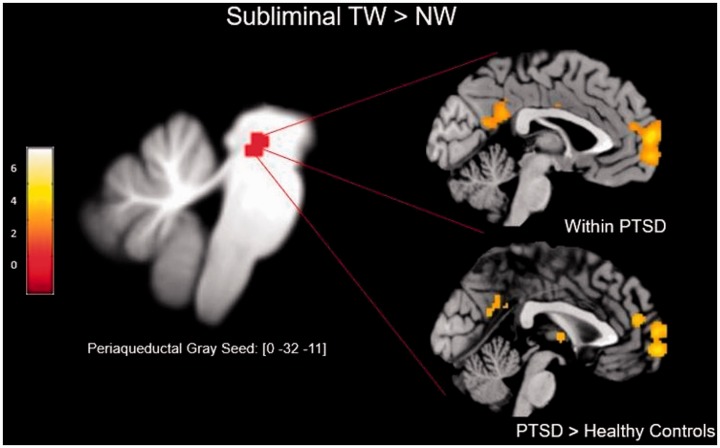
This illustration demonstrates the coordinates of significant activation as reported by Terpou et al.^29^ within SUIT-space (left). Within-subject eigenvariates were derived from the coordinates and psycho-physiological interactions were conducted at the between-group level (right). As compared to controls, the PTSD group displayed significantly greater PAG functional connectivity with multiple regions associated with the DMN (i.e., superior frontal gyrus, precuneus, angular gyrus, anterior cingulate gyrus).

Threat detection is a crucial function of the human brain with its underlying circuitry expressed within midbrain as well as cortical systems. These systems are often studied in isolation, revealing overactivation and altered functional connectivity in PTSD. To further our understanding of the effects of PTSD on threat detection and defensive response circuitry, it is critical to analyze responses to trauma-related stimuli within and across different levels of neural organization. This study revealed significant group differences in the functional connectivity of the PAG during the subliminal presentation of trauma-related stimuli. As compared to controls, individuals with PTSD displayed increased PAG functional connectivity with a range of cortical structures involved in the DMN (i.e., superior frontal gyrus, angular gyrus, precuneus) (Figure 2). Here, the DMN is recruited generally in the absence externally directed attention, when internal cognition predominates. Despite our employment of an external and subliminal stimulus, the DMN showed strong functional coupling with the PAG in the PTSD group—a novel finding of critical interest.

### Periaqueductal Gray

The DMN is a neurocognitive network engaged during processes of internally directed thought, such as mind-wandering, self-referential processing, and autobiographical memory retrieval.^36^ It is now well documented that a series of midline brain regions underlie the DMN, showing strong resting-state functional connectivity as well as robust structural connections.^62,63^ Healthy participants display increased activation and functional connectivity of the DMN in the absence of externally directed attention.^36^ By contrast, as compared to controls, individuals with PTSD exhibit reliably reduced resting-state functional connectivity of the DMN.^38,39,46,64–66^ In turn, aberrant DMN functional connectivity is thought to promote clinical disturbances of self-related processing in PTSD, which may include alterations to self-perception of the body, or emotional and perceptual experiences.^42–44^ In contrast to the reduced connectivity demonstrated at rest, the DMN has been shown to display increased functional connectivity during trauma-related processing in PTSD.^40,67^ For example, Nicholson et al.^67^ employed a thirty-minute session of neurofeedback (NFB) during fMRI that targeted the attenuation of amygdalar activity. These results demonstrated that NFB successfully shifted amygdalar connectivity from a pattern of bottom-up (pre-NFB) to a pattern of top-down connectivity (post-NFB) in participants with PTSD. In this study, bottom-up connectivity emerged in relation to functional coupling of the superficial amygdala and the PAG during the contrast of pre-NFB > post-NFB. By contrast, top-down connectivity was in relation to greater coupling between the central nucleus of the amygdala and the medial prefrontal cortex for the contrast of post-NFB >pre-NFB. Interestingly, Nicholson et al.^68^ analyzed the activation of the ICNs over the NFB paradigm and found an increase in DMN recruitment in individuals with PTSD during conditions of trauma-related stimulus exposure as compared to rest for both pre-/post-NFB. These findings corroborate our findings in that the DMN is recruited in PTSD to a greater extent during trauma-related stimulus exposure.

These results diverge markedly from the characteristics displayed by control subjects and require careful consideration. Here, it is possible that exposure to trauma-related material used in our paradigm cued the autobiographical retrieval of traumatic memories in participants with PTSD. To this end, traumatic memories are thought to be distinct in form from the aversive memories cued within the control group. For instance, some traumatic memories remain in an unprocessed state—where the cognitive, affective, and sensory components of the memory are fragmented or dissociated.^69–73^ This fragmentation of traumatic memories may result from the overwhelming affect that occurs during original encoding, thus interfering with the consolidation of the memory to long-term storage.^74–78^ In turn, the traumatic memory may remain in a state-dependent, emotionally charged form that exhibits strong perceptual priming to trauma-related cues.^72,73,79–82^ As a result, trauma-related word exposure may have triggered greater re-experiencing symptoms in individuals with PTSD as compared to controls, as exemplified, in part, by the increased state reliving scores measured by the RSDI. Whereas the precuneus and the posteromedial cortices are thought to underlie the self-referential and the visual imagery aspects of the DMN,^83,84^ the medial prefrontal cortices are thought to contribute strongly to its role in autobiographical memory.^85,86^ Importantly, both the precuneus and the superior frontal gyrus displayed greater PAG functional connectivity in the PTSD group as compared to controls. Given that the DMN displays reduced connectivity at rest in PTSD, it is possible that individuals with PTSD experience greater self-related processing in the presence of trauma-related stimuli, thus explaining the strong coupling revealed between the PAG and the DMN. In turn, this may decrease an individual’s likelihood to engage in self-related processing, promoting dissociative symptomatology. The latter supposition is supported by the multiple regression analysis, where individuals with increased state dissociation (CADSS) and avoidance scores (CAPS Criterion C) showed greater PAG functional connectivity with the middle frontal and middle temporal gyri, respectively. Taken together, these findings suggest a strong interaction between midbrain, threat-related processing systems with high-order, self-related processing systems during trauma-related stimulus processing in PTSD.

### Limitations

There are several limitations to the study. To begin, a relatively small sample was recruited, which did not permit investigation of the differences between individuals who met or did not meet the criteria for the dissociative subtype of PTSD. The subtype is distinguishable in both clinical and functional characteristics from the non-subtype of PTSD, which introduces heterogeneity to our sample.^46,47,56^ Moreover, our study follows the previous reports of group-level differences in PAG activation during subliminal threat presentation.^29^ However, the previous study did not yield significant activation of the PAG for the PTSD or control group during the subliminal display of fearful and neutral facial expressions. This did not permit the extraction of the eigenvariate for the PAG for the experimental contrast of Subliminal: FF > NF. In turn, we cannot discern whether the PAG–DMN coupling displayed in the PTSD group results from trauma-related stimulus exposure, specifically, or extends to fearful stimuli more generally. Finally, trauma-related and NWs were matched for frequency of exposure. In the event that the TWs were less common in language as compared to NWs, this may have introduced novelty effects that could increase the signal generated that are unrelated to the emotional nature of the word stimuli.

## Conclusion

These findings contribute to our understanding of self-related processing systems in PTSD. The PAG is involved in subliminal threat detection and the coordination of defensive responses and exhibits overactivation in PTSD. During the subliminal presentation of trauma-related stimuli, we extracted the seed time course of the PAG in participants with PTSD and controls to measure the functional connectivity of the structure. Strikingly, the PTSD group showed significantly greater PAG connectivity with the DMN as compared to controls. These results provide evidence for a midbrain structure exhibiting functional relatedness, and potential involvement, within large-scale cortical networks during subliminal trauma-related processing in PTSD. Given the role of the DMN in self-referential processing and of the evolutionarily-conserved function of the PAG during the execution of defensive states, functional coupling of these regions has strong clinical implications to self-referential processing systems in the presence of traumatic reminders in PTSD.

## Supplemental Material

Supplemental material for The Threatful Self: Midbrain Functional Connectivity to Cortical Midline and Parietal Regions During Subliminal Trauma-Related Processing in PTSD Click here for additional data file. Supplemental Material for The Threatful Self: Midbrain Functional Connectivity to Cortical Midline and Parietal Regions During Subliminal Trauma-Related Processing in PTSD by Braeden A. Terpou, Maria Densmore, Jean Théberge, Janine Thome, Paul Frewen, Margaret C. McKinnon and Ruth A. Lanius in Chronic Stress
