# Supplementary material for: The Threatful Self: Midbrain Functional Connectivity to Cortical Midline and Parietal Regions During Subliminal Trauma-Related Processing in PTSD
Source: Chronic Stress (Thousand Oaks). 2019 Sep 5;3:2470547019871369. doi: 10.1177/2470547019871369 (PMC7219912; doi:10.1177/2470547019871369)
Supplement: Supplemental material for The Threatful Self: Midbrain Functional Connectivity to Cortical Midline and Parietal Regions During Subliminal Trauma-Related Processing in PTSD [file Supplemental_Material.pdf]

Supplemental Information:

Methods and Materials

Whole-Brain Functional Data Preprocessing:

All images were analyzed using Statistical Parametric Mapping (SPM12, Wellcome Trust Centre for Neuroimaging, London, UK: http://www.fil.ion.ucl.ac.uk/sp) within MATLAB 9.2 (R2017a, Mathworks Inc., MA). Firstly, functional images for each participant were realigned to the first volume of the session to correct for movement during scanning. During realignment, a mean functional image was created for each subject, which was used to co-register the T1-weighted anatomical image so to align subject-specific BOLD signals within their anatomical space. All volumes were spatially normalized (2 x 2 x 2 mm^3) to an EPI in MNI space through the application of a deformation matrix. At this time, an ART regressor, which accounts for effects of movement and global signal correction (version 2015-10; Gabrieli Lab, McGovern Institute for Brain Research, Cambridge, MA), was generated for subliminal and supraliminal presentation sessions. Default thresholds for the outlier detection in the ART regressor were selected (global signal threshold = 9.0 mm, absolute subject motion threshold = 2.0 mm, rotational threshold = .05 mm, scan-to-scan subject motion = 2.0 mm, and scan-to-scan subject rotation = .02 mm). Finally, functional images were then smoothed via a three-dimensional isotropic 8 mm full-width at half-maximum Gaussian kernel and a high-pass filter was applied to reduce data low-frequency noise.

SUIT-Space Functional Data Preprocessing:

To improve the normalization procedure and receive a clearer depiction of midbrain, lower brainstem, and cerebellar activation, data were also normalized to the spatially unbiased infra-tentorial template (SUIT).[73,74] The SUIT toolbox offers a high-resolution atlas template of the

cerebellum and brainstem with improved voxel-by-voxel normalization of fMRI. The SUIT toolbox functions on SPM12 within MATLAB 9.2 and contains several preprocessing steps. Firstly, anatomical images were reoriented in SPM where the horizontal plane was defined approximately according to the AC-PC line. Secondly, functional images were reoriented to correspond to the reoriented anatomical image. Thirdly, subject-specific functional volumes were realigned to the first volume of each session to correct for movement in the scanner and then resliced to a voxel size of 2 x 2 x 2 mm^3. At this time, six realignment parameters for changes in motion across the different planes and an ART regressor for global movement correction were saved for each participant. Fourthly, subject-specific brainstem and cerebellum were isolated and cropped from the T1-weighted anatomical images in order to focus on the infra-tentorial structures of interest. Fifthly, individual cropped anatomical images of the brainstem and cerebellum were normalized into the SUIT atlas template. During this step, a subject-specific transformation matrix was generated for the linear part of the normalization that deforms each cerebellum to provide optimal correspondence to the SUIT template. Sixthly, functional volumes were resliced into SUIT-space in order to align functional images with the SUIT-normalized anatomical images by applying the subject-specific transformation matrix. Lastly, a three-dimensional isotropic 4 mm full-width at half-maximum Gaussian kernel was applied to each set of SUIT-resliced functional data to smooth the data in accordance with previous methods using SUIT preprocessing measures.
